# ID 57 - ATS no Inca: integração da prevenção e vigilância, assistência, ensino e pesquisa

**DOI:** 10.5327/2237-9622.2025.v34s1.57

**Published:** 2025-11-25

**Authors:** Ana Paula Rodrigues Siqueira, Rodrigo Saar da Costa, Isabel Cristina de Almeida Santiago, Laura Augusta Barufaldi

## Abstract

**Introdução:**

O Instituto Nacional de Câncer (Inca), protagonista da Política Nacional de Prevenção, Diagnóstico e Tratamento do Câncer (Lei n.º 14.758, de 19 de dezembro de 2023), e formulador de diretrizes para o controle do câncer, detém demandas específicas de análises de avaliações de tecnologias em oncologia que atendam à instituição e à sociedade.

Nesse sentido, o Núcleo de Avaliação de Tecnologias em Saúde do Inca foi instituído por meio da Portaria n.º 249, de 08 de agosto de 2011. Atualmente, designada como Divisão de Avaliação de Tecnologias em Saúde (Dats), tem como missão promover a produção de conhecimento científico para contribuir na construção de um processo decisório eficiente para a utilização de tecnologias em saúde, voltadas para o controle do câncer.

Em 2024, foi desenvolvido o projeto intitulado ATS no Inca, com a proposta de implementação de ações pela Dats, que subsidiem a instituição no contexto da gestão em avaliações de tecnologias em saúde na área da oncologia.

A iniciativa tem como finalidade fomentar a articulação de demandas de avaliação de tecnologias em saúde em oncologia com as áreas de assistência, ensino e pesquisa do Inca, definindo temas prioritários e em consonância com a Política Nacional de Prevenção, Diagnóstico e Tratamento do Câncer. O projeto será exposto em formato de apresentação oral utilizando recursos visuais e eslaides.

**Método:**

Apresentação de metas e de objetivos: o objetivo do projeto é que o Inca seja, além de referência nacional, um polo de descentralização do conhecimento em ATS em oncologia, capacitando profissionais e subsidiando a instituição na tomada de decisão e potenciais articulações no processo de construção de um sistema de saúde voltado para o paciente oncológico em um contexto sustentável e equânime.

Apresentação de etapas: na elaboração do estudo analítico do projeto foram planejadas três etapas a serem executadas ao longo do período. A primeira etapa será a descentralização do conhecimento acerca da avaliação de tecnologias em saúde dentro do Inca, a segunda etapa será a definição de temas prioritários em oncologia para desenvolvimento de estudos de ATS, bem como estabelecimento de fluxo institucional de entrada de demanda de análise e a terceira e última etapa será a produção técnico-científica oriunda das avaliações demandadas.

Análise Gerencial e execução: o projeto encontra-se em fase de execução, tendo a primeira etapa concluída. Os materiais elaborados na fase de descentralização do conhecimento foram: três infográficos, uma publicação no Informe Inca, um vídeo institucional e um Podcast.

Resultados esperados: os resultados esperados desse projeto compreendem a promoção de um ambiente de permanente intercâmbio de conhecimento em ATS, o fortalecimento do capital intelectual e a capacidade institucional quanto à ATS, propor ferramentas, iniciativas e sistemáticas para viabilizar e aprimorar a capacidade de realização de ATS e disseminar resultados.

